# The diagnostic accuracy of intraoperative differentiation and delineation techniques in brain tumours

**DOI:** 10.1007/s12672-022-00585-z

**Published:** 2022-11-10

**Authors:** Laura Van Hese, Steven De Vleeschouwer, Tom Theys, Steffen Rex, Ron M. A. Heeren, Eva Cuypers

**Affiliations:** 1 Division of Mass Spectrometry Imaging, Maastricht MultiModal Molecular Imaging (M4I) Institute, Maastricht University, Universiteitssingel 50, 6229 ER Maastricht, The Netherlands; 2 Neurosurgery Department, University Hospitals Leuven, 3000 Leuven, Belgium; 3 Laboratory for Experimental Neurosurgery and Neuroanatomy, Department of Neurosciences, Leuven Brain Institute (LBI), 3000 Leuven, Belgium; 4 Department of Anaesthesiology, University Hospitals Leuven, 3000 Leuven, Belgium; 5 Department of Cardiovascular Sciences, KU Leuven, 3000 Leuven, Belgium

**Keywords:** Brain tumour, Intraoperative characterisation, Tumour margin delineation, Literature review

## Abstract

Brain tumour identification and delineation in a timeframe of seconds would significantly guide and support surgical decisions. Here, treatment is often complicated by the infiltration of gliomas in the surrounding brain parenchyma. Accurate delineation of the invasive margins is essential to increase the extent of resection and to avoid postoperative neurological deficits. Currently, histopathological annotation of brain biopsies and genetic phenotyping still define the first line treatment, where results become only available after surgery. Furthermore, adjuvant techniques to improve intraoperative visualisation of the tumour tissue have been developed and validated. In this review, we focused on the sensitivity and specificity of conventional techniques to characterise the tumour type and margin, specifically fluorescent-guided surgery, neuronavigation and intraoperative imaging as well as on more experimental techniques such as mass spectrometry-based diagnostics, Raman spectrometry and hyperspectral imaging. Based on our findings, all investigated methods had their advantages and limitations, guiding researchers towards the combined use of intraoperative imaging techniques. This can lead to an improved outcome in terms of extent of tumour resection and progression free survival while preserving neurological outcome of the patients.

Gliomas are central nervous system tumours that arise from glial cells such as astrocytes, oligodendrocytes and ependymal cells. Patient diagnosis and treatment response strongly depend on the different glioma subtypes and make accurate diagnosis imperative. Therefore, reliable intraoperative detection and characterisation of tumour tissue plays an essential role in determining diagnosis, in optimizing the surgical procedure and in the postoperative treatment plan of the patient.

Over the last decades, surgical techniques in brain lesions have strongly evolved. Improved imaging techniques and brain mapping techniques, and advances such as endoscopic surgery, microscopic surgery, and stereotactic biopsies play a crucial role in diagnosis and optimal management of the patients with gliomas. Several studies have shown that the extent of resection is positively correlated with progression free survival and quality of life [1]. However, due to the infiltrative behaviour of glioma, differentiation of tumour tissue from adjacent normal functioning brain remains a challenge. While it is well known that tumour cells spread far beyond the radiologically visible borders, post-operative magnetic resonance imaging (MRI) is still considered the reference to assess the presence of residual tumour near the resection cavity. Several technologies were recently introduced to help the neurosurgeon identify tumour tissue intraoperatively. In this review, we discuss the diagnostic accuracy of standard as well as experimental techniques for the intraoperative differentiation and delineation of gliomas.

## Fluorescent guided surgery

Fluorescent guided surgery (FGS) has provided neurosurgeons with improved intraoperative visualisation of malignant brain tumours. Fluorophore agents include 5-aminolevulinic acid (5-ALA), fluorescein and indocyanine green (ICG) and have been commonly studied in high grade glioma (HGG). The fluorescence emitted by these fluorophores helps the surgeon distinguish the tumour core from the infiltrating tumour margin and typically emits light in the visible and near infrared spectrum. The corresponding landmark studies are summarised in Table 1.

**Table 1:** An overview of studies investigating diagnostic accuracy of fluorescent-guided surgery in brain tumours

Study	Method	# Patients	Pathology	Golden standard	Sensitivity (%)	Specificity (%)
Valdes et al. [22]	5-ALA	14	Brain tumours	Conventional fluorescence imaging	84.0	92.0
Panciani et al. [33]	5-ALA	23	GBM	Pathology	91.0	89.0
Coburger et al. [15]	5-ALA	45	HGG and metastasis	Pathology	91.0	80.0
Rey-Dios et al. [5]	Fluorescein	6	HGG	Pathologist examination	79.0	100
Diaz et al. [3]	Fluorescein	12	GBM	Pathologist examination	82.2	90.9
Neira et al. [4]	Fluorescein	32	GBM	Pathologist examination	75.6	75.0
Catapano et al. [6]	Fluorescein	23	HGG	Pathologist examination	84.6	95.0
Zhang et al. [7]	Fluorescein	38	HGG	Pathologist examination	94.4	88.6
Lee et al. [28]	Indocyanine green	15	Gliomas	Pathology	98.0	45.0
Cho et al. [29]	Indocyanine green	36	HGG	Pathology	97.0	56.0
Shen et al. [30]	Indocyanine green	23	Gliomas	Convolutional neural network	93.8	82.2
				Pathology	91.2	60.6

*5-ALA* 5-aminolevulinic acid, *GBM* glioblastoma multiforme, *HGG* high grade glioma

### Fluorescein

The sodium salt of fluorescein emits a yellow-green fluorescence under white light (maximum absorbance of 494 nm and emission maximum of 521 nm) and has been used to localise gliomas during open biopsy since 1948 [2]. Fluorescein, essentially an intravascular dye, is injected intravenously and has the capability to penetrate brain areas with altered blood–brain barrier (BBB) [3]. As a result, fluorescein-based fluorescence is not specific for glioma cells. Multiple studies evaluated the sensitivity and specificity of fluorescein guided resection of gliomas [3–8]. They found a median sensitivity and specificity of 82.2% and 90.9%, respectively. In 2018, a phase II trial, in 57 patients with HGG, using a dedicated fluorescence filter in the surgical microscope (FLUOGLIO) investigated the efficacy of fluorescein in HGG [9]. Here, 82.6% of the patient underwent gross total resection and fluorescent tissue at the infiltrating tumour margin was identified with 80.8% sensitivity and 79.1% specificity. In line with this, Neira et al*.* also considered the utility of fluoresceine to classify biopsies from the tumour margin using a quantitative measure of the fluorescence levels [4]. The sensitivity (69.4%) and specificity (66.7%) clearly decreased. Furthermore, in 2020, Manoharan et al*.*, studied the correlation of the fluorescence intensity at the tumour margin and the presence of residual contrast-enhancing tumour on post-operative MRI [10]. They found a sensitivity of 66.7% and specificity of 75%, in agreement with the studies of Acerbi et al*.* and Neira et al. even if the time frame and kinetics of dye administration used, differed in both studies [4, 9]. It can be concluded that further validation of this technique is needed in combination with appropriate post-operative imaging and intraoperative quantitative analysis of the fluorescence levels of the resected tissue [10]. Currently, in practice, this technique is predominantly used for vascular neurosurgery since fluorescein as intravascular dye in the patient’s blood contaminates the operative field during brain tumour resection whenever blood is blurring the surgical site.

### 5-Aminolevulinic acid

5-ALA is the precursor of the fluorescent protoporphyrin IX (PpIX) in the heme biosynthesis pathway. PpIX is not further metabolised and accumulates intracellularly in tumour cells of epithelial and mesenchymal origin and fluoresces bright red when exposed to violet-blue light, while normal brain tissue remains dark [11]. This endogenous fluorescent PpIX absorbs light at 405 nm and emits fluorescence with a main peak centred at 634 nm [11, 12]. Stummer et al. investigated the application of 5-ALA in newly diagnosed malignant gliomas [13]. They showed a significant improvement in complete resection of contrast-enhancing tumours in the 5-ALA group compared to visible light group (65% vs. 36%, respectively). Moreover, the 5-ALA group showed a longer progression-free interval and median survival times. Up to now, numerous studies already showed the high sensitivity and positive predictive value (PPV) of 5-ALA in new and recurrent HGG [11, 14, 15]. Two meta-analyses that pooled the data on sensitivity and specificity of 5-ALA for HGG showed sensitivity data ranging from 81 to 83% and specificity data ranging from 89 to 90% [16, 17]. However, from the microscopic point of view, the sensitivity still remains too low for guiding resections at the infiltrating tumour margin simply because PpIX accumulation is typically below the limit of detection of the conventional surgical microscopes. In the infiltrative margins of diffuse high-grade gliomas PpIX fluorescence decreases and is therefore difficult to consider in the proximity of non-fluorescent normal brain tissue. A recent study showed that the residual median cell density in non-fluorescent tissue was still 13% [18]. On the other hand, in low grade glioma (LGG) the use of 5-ALA remains limited, since visible fluorescence is observed in only 10 to 20% of patients using fluorescence microscopy [19, 20]. Furthermore, evaluation of the shade of fluorescence strongly depends on the expertise of the neurosurgeon and is difficult to quantify [21]. Here, more sensitive technologies, such as confocal microscopy and spectroscopy are developed to measure the absolute concentrations of PpIX fluorescence more accurately [22–24]. However, for the resection of malignant gliomas, 5-ALA is currently considered the gold standard due to its high efficacy and safety.

### Indocyanine green

Indocyanine green (ICG) is an imaging agent that operates in the first near infrared window (NIR-I, 700–900 nm) with an excitation wavelength of 740–800 nm, and emission wavelength in the 800–860 nm [25]. Like fluorescein, it penetrates areas of BBB disruption, after intravenous injection. Next to its conventional use as an angiographic agent, second-window-ICG (SWIG) has been demonstrated recently in labelling tumour tissue. Here, ICG is administered to the patient the day prior to surgery in a higher dose. This allows accumulation in the extracellular compartment of the tumour environment (and perilesional oedema) [26, 27]. In 2016, the first study in 15 patients with gliomas was published [28]. They demonstrated a 98% sensitivity and 45% specificity for SWIG to identify tumour tissue, compared to a 84% sensitivity and 80% specificity under white-light. Later, the same group, showed that SWIG has a 97% sensitivity and 56% specificity for margin specimens in contrast-enhancing tumours, compared to 78% sensitivity and 100% specificity under white light [29]. At last, a recent study developed fluorescent imaging coupled to an artificial intelligence technique [30]. This technique, based on deep learning, was able to efficiently distinguish tumour and non-tumour during the course of surgery with 93.8% sensitivity and 82.2% specificity. NIR fluorescence typically shows a higher sensitivity, but lower specificity compared to the other fluorescent agents. However, combined with deep learning techniques, comparable specificity and even higher sensitivity was demonstrated [30]. Furthermore, NIR window II (NIR-II, 1000 to 1700 nm) fluorophores show a more accurate detection, better contrast and lower tissue absorption compared to NIR I and the visible spectrum. So far, these promising results were mostly seen in preclinical applications using small animal models [31]. However, recently, its performance was tested for the first time in clinic in 7 patients. They used a multispectral fluorescence imaging instrument together with an image fusion method to precisely block the vessels around the tumour, leading to a dramatically decreased blood loss [32]. Future clinical studies should further confirm the increased sensitivity and specificity of these fluorophores to distinguish between normal and tumoral tissue in human gliomas.

## Intraoperative image guidance

Significant improvements have been made in the development of imaging technologies that provide the surgeon with a better insight into the location and extent of the brain tumour. Currently, tumour delineation is established using imaging modalities such as intraoperative MRI (iMRI), neuronavigation and intraoperative ultrasound (iUS). Pre- and intra-operative images link the anatomical landmarks and can further define the surgical approach. Table 2 summarizes the landmark studies for these modalities.

**Table 2:** Studies investigating diagnostic accuracy of intraoperative image guidance in brain tumours

Study	Method	# Patients	Pathology	Golden standard	Sensitivity (%)	Specificity (%)
Panciani et al. [33]	Neuro-navigatin	23	GBM	Pathology	57.8	57.4
Kubben et al. [36]	iMRI	10	GBM	Pathology	49.0	100
Coburger et al*.* [15]	iMRI	34	HGG	Pathology	41.0	70.0
Coburger et al. [37]	iMRI	20	GBM	Pathology	55.1	73.7
Hesselmann et al. [38]	iMRI	68	Primary GBM	Pathology	96.0	75.0
			Recurrent GBM	Pathology	94.0	57.0
Gerganov et al. [41]	iUS	11	LGG	postoperative MRI	80.0	83.3
Arlt et al. [42]	iUS	50	LGG and HGG	postoperative MRI	85	28
Sweeney et al. [43]	iUS	260	LGG and HGG	postoperative MRI	50.8	100
Munkvold et al. [39]	iUS	144	LGG and HGG	postoperative MRI	46.0	85.0

*GBM* glioblastoma multiforme, *HGG* high grade glioma, *iMRI* intraoperative magnetic resonance imaging, *iUS* intraoperative ultrasound, *LGG* low grade glioma

### Neuronavigation

Neuronavigation systems, or frameless stereotactic systems, allow to transpose imaging information to the operative field by showing the tip of a pointer, or the focus point of the microscope in a preoperatively obtained image. Here, a relationship between the image space and patient’s brain anatomy has to be established. Currently, this relationship is strongly influenced by intra-operative tissue displacement or brain shift. In 2012, Panciani et al*.* investigated the effectiveness of neuronavigation and showed a sensitivity and specificity of 57.8% and 57.4% when used alone [33]. However, the combined use of 5-ALA and neuronavigation led to an increase in the extent of resection and in the sensitivity of 5-ALA. Notably, neuronavigation for the resection of brain tumours is most useful when combined with other brain-mapping techniques. The integration of iMRI and iUS can be applied in order to update the navigation system during the course of surgery [34].

### Intraoperative MRI

Over the past decade, iMRI has been increasingly used during the resection of brain tumours. While conventional neuronavigation proved to be less reliable after the surgery has started, iMRI can be re-registered during surgery, to account for brain shift. Furthermore, high-field strength iMRI might visualise remaining contrast enhancement at the margin of resection, indicating residual tumour. However, iMRI requires an expensive set-up and is usually only used in a selected patient population and less often in HGG [35]. Currently, few reports have discussed the diagnostic accuracy of iMRI in glioma surgery. In 2012, Kubben et al*.* found a 49% sensitivity and 100% specificity for the detection of HGG [36]. Furthermore, Coburger et al*.* evaluated the sensitivity and specificity of iMRI in two comparative studies. First, in a comparison with 5-ALA, iMRI showed a sensitivity and specificity of 41% and 70%, respectively, compared to a 85% sensitivity and 43% specificity for 5-ALA [15]. Second, linear array intraoperative ultrasound (lioUS) was evaluated compared with high field iMRI and conventional sector array ultrasound (cioUS). Here, iMRI showed a 55.1% sensitivity and a 73.7% specificity [37]. At last, Hesselmann et al*.*, showed that iMRI is effective for the detection of remaining tumour tissue after resection, with a sensitivity of 95% and specificity of 69.5% [38]. Furthermore, iMRI can be more useful when combined with other techniques such as neuronavigation and fluorescent-guided surgery, to allow a theoretically better preservation of white matter connections.

### Intraoperative ultrasound

iUS has been frequently used as a complementary technique to overcome some of the limitations of image guided resection of gliomas. This techniques allows for inexpensive and real-time intraoperative imaging with the possibility of integration into a neuronavigation system for simultaneous navigation in the preoperative MRI images and intraoperative 3-dimensional US [39]. Gerganov et al*.* compared 2D US with high-field iMRI in 26 patients and showed that iMRI was able to reveal 80.8% of tumour remnants, whereas 2D US was not effective in detecting tumour remnants smaller than 1 cm [40]. Furthermore, they evaluated the quality and reliability of 2D US for the removal of LGG. Here, iUS allowed visualisation of the tumour margin in 81.8% of patients, while iMRI allowed margin detection in 90.9% [41]. In 2008/2009, 3D reconstructed and contrast-enhanced ultrasound (CEUS) made its introduction for the neurosurgical resection of brain tumours. Different from MRI where contrast agents show areas where the blood–brain barrier is damaged, US contrast agents show tumour vascularisation. In a study of Arlt et al*.*, these areas of contrast agent uptake showed high sensitivity (85%) but low specificity (28%) due to the infiltrative nature of malignant brain tumours [42]. In 2018, a large study of 260 patients showed a high rate of false negatives (25%) for iUS in the determination of complete glioma resection, probably due to the highly invasive nature of these HGG [43]. Furthermore, Munkvold et al*.* showed a relatively lower specificity (77%) in HGG compared to LGG (94%). The specificity decreased even more in patients who had previously undergone radiotherapy (50%). This lower specificity in HGG might be partially explained by the oedema surrounding HGG lesions, and the often heterogeneous nature of these tumours. Here, fluorescent guided surgery might be more appropriate for the intraoperative diagnostics in HGG. In contrast, in LGG most studies confirm that the diagnostic properties of iUS are good [39].

## Experimental techniques

### Mass spectrometry

Mass spectrometry imaging (MSI) uses either a solvent (e.g., DESI), a laser (e.g. MALDI) or ion beam (e.g. SIMS) to desorb and ionize molecules from a tissue surface. These ionized molecules are sent to a mass spectrometer to measure their mass to charge ratio and relative abundance. A mass spectrum is acquired for every analysis spot. The spatial distribution of each compound on the tissue surface is shown and allows to look for changes in relative abundance of ions between healthy and tumoral tissue. This technique can analyse nearly all types of biomolecules and biological species with a substantial sensitivity, specificity, making it an attractive tool in biomedical research. However, up to now, the intraoperative use is still limited due to the need of freezing and cryosectioning before analysis. Table 3 provides an overview of the most important studies using experimental techniques in the characterisation of brain tumours.

**Table 3:** An overview of studies evaluating the sensitivity and specificity of experimental techniques for the characterisation of brain tumours

Study	Methods	# Patients	Pathology	Golden standard	Sensitivity (%)	Specificity (%)
Jarmusch et al. [47]	DESI-MS	74	Glioma	Pathology	97.4	98.5
Jarmusch et al. [44]	DESI-MS	39	Glioma	Pathology	91.4	98.7
Pirro et al. [48]	DESI-MS	10	Glioma	Pathology	93.0	83
Brown et al. [49]	DESI-MS	49	Diffuse glioma	Pathology	63	83
Balog et al. [60]	REIMS	11	Glioma	Pathology	100	100
Haapala et al. [63]	DMS	28	Glioma and meta’s	Pathology	78	89
Van Hese et al. [61]	REIMS	36	LGG	Pathology	99.2	–
			GBM		95.3	–
Livermore et al. [67]	Raman spectroscopy	11	LGG and HGG	Pathology	96.0	99.0
Desroches et al. [66]	Raman spectroscopy	10	Glioma	Pathology	84.0	89.0
Jermyn et al. [65]	Raman spectroscopy	17	LGG and HGG	Pathology	93.0	91.0
Kalkanis et al. [64]	Raman spectroscopy	40	GBM	Pathology	96.0	100
Urbanos et al. [74]	Hyperspectral imaging	12	HGG	Pathology	8–98	72–100
Manni et al. [73]	Hyperspectral imaging	9	GBM	Pathology	68.0	98.0
Fabelo et al. [72]	Hyperspectral imaging	6	GBM	Pathology	49–88	88–100
Miller et al. [77]	Molecular targeted NIR	1	GBM (low dose)	Pathology	73.0	66.3
		1	GBM (high dose)	Pathology	98.2	69.8

*DESI-MS* desorption ionisation mass spectrometry, *DMS* differential mobility spectrometery, *GBM* glioblastoma multiforme, *HGG* high grade glioma, *LGG* low grade glioma, *REIMS* rapid evaporative ionisation mass spectrometer

#### Desorption electrospray ionisation mass spectrometry

Desorption electrospray ionisation mass spectrometry (DESI-MS) is an ambient ionisation technique that provides molecular information directly from the tissue sample at atmospheric pressure and usually require no sample preparation. In 2012, Eberlin et al*.* showed the ability of DESI-MS to classify gliomas of different subtypes, grades and cell concentration based on the lipid profile of these primary human brain tumours. Using this technique, the spatial information is retained. This allows validation of the results with histopathological evaluation. The molecular information obtained from DESI-MS provides accurate diagnosis and a valuable insight into tumour cell concentrations to define the tumour margin [44, 45]. Eberlin et al*.* first described a lipidomic based approach for the classification of gliomas with a recognition capability of more than 99% and a cross validation of more than 97% [45]. It is noteworthy that this classification model was built using only glioma subtypes; astrocytoma, oligodendroglioma and oligoastrocytoma and their different grades. They developed a classification model to distinguish between gliomas and meningiomas using DESI-MSI with 99.7% cross validation [46]. Furthermore, the use of tissue smears was evaluated and allowed intraoperative decision making and pathological evaluation. In line with the results of Eberlin et al*.*, a high sensitivity and specificity of more than 90% was found [44, 47]. Furthermore, the act of smearing did show no significant alterations in the observed lipid or metabolite profiles [47]. In 2016, this technique was tested in the operating room on neurological tissue smears from 10 subject who underwent glioma resection. DESI-MS allowed detection of glioma and the estimation of the percentage of tumour cells at the invasive margin with 93% sensitivity and 83% specificity [48]. Recently, DESI-MSI was evaluated for the intra-operative detection of the isocitrate dehydrogenase mutation, the estimation of tumour cell infiltration and the disease status. Here, the disease status was predicted with a 63% sensitivity, 83% specificity and 74% accuracy [49]. DESI-MS provides results within a few minutes and is applicable to every surgical procedure without the need for procedural alterations. The use of tissue smears eliminates the need to freeze and cryosection the samples prior to DESI-MS analysis. Validation by a pathologist is possible by staining and evaluating the same tissue smear.

#### Matrix-assisted laser desorption ionisation

Matrix-assisted laser desorption ionisation-time of flight (MALDI-TOF) mass spectrometry is a versatile analytical tool that was first described by Caprioli et al*.* [50]. This technique shows high sensitivity and specificity with morphological information about the spatial distribution of biomolecules in cells and tissues. MALDI has been frequently used for the analysis of human tissue samples, including biomarker discovery and diagnosis [51, 52]. A tissue section or smear is first covered by a layer of matrix material, prior to the introduction into the vacuum chamber of the mass spectrometer for analysis. Depending on the matrix that was applied, analytes ranging from proteins to lipids and metabolites can be detected by MALDI-MS in a few minutes. In 2010, Agar et al*.* adapted MSI for clinical applications to accurately evaluate brain tumour specimens at the time of surgery. They built a preliminary classifier and showed the ability of MALDI-MSI to differentiate between meningioma, non-tumour brain and glioma [53]. Furthermore, MALDI-MSI was used to evaluate peptides in a set of grade III gliomas. Here, molecular discrepancies were identified between the histological annotation of the neuropathologist and the molecular analysis done by MALDI-MSI [54]. These results suggest that MALDI-MSI might improve histopathological diagnosis by highlighting the spatial molecular heterogeneity in gliomas. Longuespée et al*.* showed a rapid approach (less than 5 min) to detect d-2-hydroxyglutarate based on MALDI-MSI [55]. This supports the possibility to use this technique as a tool for helping the neuropathologists to test for multiple markers with diagnostic relevance. In paediatric brain tumours, the molecular profiles of tumour specimens were analysed to identify potential markers to distinguish medulloblastoma and pineoblastoma. Here, MALDI-MSI also showed to be able to classify between these two tumour types based on differences in the lipid profiles [56]. Furthermore, to better understand the mechanisms by which medulloblastoma metastasis occur, Paine et al*.* performed MALDI-MSI experiments on a mice model of human medulloblastoma [57]. In line with the previous reported studies, low abundance, spatially-heterogeneous lipids were found to be associated with the tumour development. Recently, different human glioblastoma cell lines were identified using MALDI-MSI [58]. This study showed the ability of MALDI-MSI for the detection of single cells in glioblastoma (GBM) tumours and therefore demonstrates the added value this technique could have in the digital pathology of brain tumours. We can conclude that MALDI-TOF is a rapid and inexpensive procedure that could improve the classification of brain tumours. However, clinical trials on glioma patients are required to further validate this technique.

#### Diathermy smoke

Electro-cauterisation devices are commonly used in the surgical resection of many brain tumours, in order to achieve haemostasis and improve the accuracy of dissection. Here, a major by-product of their use is smoke that results from evaporating tissue debris during resection. This smoke carries molecular information about the resected tissue (e.g. biomarkers and metabolites) [59]. The ambient ionisation technique, rapid evaporative ionisation mass spectrometry (REIMS), connects an electrosurgical knife to a mass spectrometer (the iKnife) and thereby converts tissue components into gas-phase ionic species that can be analysed by the mass spectrometer. A first study in brain showed accurate identification with 100% sensitivity and specificity [60]. Recently, Van Hese et al*.* validated the REIMS technique for the molecular characterisation of HGG and LGG and determined the diagnostic accuracy and sensitivity of REIMS to detect tumour cells in the tumour margin of both HGG and LGG. Here, astrocytomas and oligodendrogliomas (WHO grade II) were separated from normal brain with a 96% correct classification rate. Furthermore, GBM tissue was differentiated from the surrounding normal brain with 99.6% sensitivity [61]. Both DESI-MS and REIMS characterize these tumours based on their lipid profile using different ionisation methods. However, the analysis time of REIMS takes only seconds, compared to DESI taking about 3 min before feedback to the surgeon [48]. Furthermore, REIMS provides instant feedback to the surgeon as it can be performed in-vivo, whilst this is not possible for DESI. These two techniques provide complementary information in the characterisation of brain tumours. DESI-MS allows the acquisition of images of the tissue providing a correlation of chemical and spatial information. However, this spatial information is lost when tissue smears are used for DESI-MS. When using tissue sections for DESI-MS, the spatial distribution of molecular markers detected by REIMS can be shown. Apart from the electrosurgical knife, a mass spectrometer has also been coupled with a Cavitron Ultrasonic Surgical Aspirator (CUSA). In the limited amount of samples (n = 11), an identification performance for GBM and normal brain of 100% was found [62]. At last, the surgical smoke was analysed using differential mobility spectrometry (DMS). Here, a high-frequency electric field is used to break molecule clusters to create additional information on the cluster strengths. They showed an overall classification accuracy of 83% with a 78% sensitivity and 89% specificity to separate the control samples from tumour [63].

### Raman spectroscopy

Raman spectroscopy is a laser-based technique that gives spectral tissue features based on the molecular signature of the tissue that is analysed. This technique differentiates molecules in a non-destructive way resulting from the inelastic scattering of incident light. Kalkanis et al*.* showed a 99.5% and 97.8% accuracy to distinguish normal grey matter, necrosis and GBM, respectively [64]. However, due to freeze artifacts in the investigated tissue samples, it was difficult to visually confirm the presence of normal viable tissue or neoplastic tissue. Therefore, the Raman-based probe technique was tested in patients during surgical resection of gliomas. Jermyn et al*.* demonstrated that Raman spectroscopy can distinguish cancer cell-invaded brain from 3 normal brain tissue with a sensitivity and specificity of more than 90% [65]. In another study, the same group showed that this technique could intra-operatively detect previously undetectable invasive tumour cells in patients with gliomas (WHO grade 2–4). Necrosis was distinguished from normal vital tissue with an accuracy of 87% and a sensitivity and specificity of 84% and 89%, respectively [66]. Furthermore, the false-negative rate was evaluated and showed a detection threshold of approximately 15% cancer cells. Recently, Livermore et al*.* compared the ex-vivo performance of Raman spectrometry with 5-ALA in predicting infiltration versus normal brain tissue at the invasive margin. Here, Raman spectroscopy showed to be significantly superior to 5-ALA, with 24% accuracy, 7% sensitivity and 100% specificity [67]. Although the higher detection rate of Raman spectroscopy in this study, only a limited number of cases were investigated ex-vivo. Furthermore, Hollon et al*.* demonstrated how combining stimulated Raman histology and deep conventional neural networks can rapidly predict intraoperative tumour diagnosis. In a multicenter study of 278 patients they showed an overall accuracy of 94.6% for the stimulated Raman histology group compared to a 93.9% for the conventional H&E histology arm, exceeding their threshold for noninferiority (> 91%) [68]. Real-time in-vivo Raman spectroscopy is a developing tool with the potential for integration in the surgical workflow of brain tumours. However, significant challenges still need to be solved, such as reduced performance due to blood and fluid, loss of signal and noise from surrounding lights. Therefore, a close collaboration between surgeons and clinical spectroscopists will be crucial to develop an intra-operative device that is easy-to-use and interpret and has the required accuracy [67].

### Hyperspectral imaging

Hyperspectral imaging is a wide-field, non-contact, non-ionising sensing technique that unlike other images covers a wide range of the electromagnetic spectrum (400 to 2500 nm) [69]. This technique combines the conventional imaging and spectroscopy to simultaneously obtain spatial and spectral information of the investigated tissue. Therefore, a large number of spectral bands per pixel in the image are captured and generated a high amount of information. Depending on the type of hyperspectral camera, these images can be composed of thousands of spectral channels, that all require high-performance computing. Currently, the large amount of data represents one of the main challenges of hyperspectral imaging. In brain tumours, quantitative and qualitative hyperspectral imaging has been evaluated for the delineation of tumour boundaries. Fabelo et al*.* tested an algorithm in a training set of patients with GBM and reached an accuracy of 77%. However, they didn’t manage to improve the tumour classification accuracy (42%) [70–72]. Recently, a hybrid deep learning framework was evaluated to quantitatively classify brain and tumour tissue in an in-vivo hyperspectral brain dataset. Here, an overall accuracy of 80% was obtained to classify tumour, healthy tissue and blood vessels [73]. However, using the same algorithm, Urbanos et al*.* only achieved an overall accuracy of 60% [74]. The difference in accuracy results between these studies might be attributed to the amount of classes for classification. Manni et al*.* used four classes (e.g. normal, tumour, blood vessels and background) while Urbanos et al*.* used five classes: healthy tissue, tumour, venous blood, arterial blood and dura mater.

### Molecular targeted near-infrared fluorescence imaging

As already discussed in fluorescent guided surgery, some limitations, such as non-specific fluorescence and unwanted side effects, are still associated with most currently used fluorophores. However, over the last years new strategies for fluorescent guided surgery have been developed (e.g., the investigation of novel fluorescent agents and probes). An interesting new approach targets known tumour-specific ligands, such as EGFR. These fluorophores accumulate in a specific part overexpressed in the tumour and can be employed to visualise the tumour intra-operatively [75, 76]. Epidermal growth factor receptor (EGFR) protein is highly expressed in the majority of HGG and therefore a promising target. Miller et al. performed the first In-human trial using a conjugate of cetuximab, a monoclonal antibody that inhibits EGFR, and IRDye 800 CW, a NIR dye that emits at 805 nm. They investigated two patients (one low and one high dose) and distinguished tumour from normal tissue with a sensitivity of 73% and specificity of 66.3% in the low dose patient, and of 98.2% sensitivity and 69.8% specificity in the high dose patient [77]. Future research should investigate different fluorophores, targets, and possible combinations with other fluorescent agents (e.g., 5-ALA and EGFR-targeting fluorophore), to determine the administration schedule and optimal dose [78, 79]. Currently, numerous fluorescent probes are still under early clinical and preclinical investigation [75].

## Strengths and limitations

In Table 4 an overview is provided of the strengths and weaknesses of the different diagnostic techniques. 5-ALA showed to be a reliable and immediately available intraoperative marker. In contrast, ICG as a NIR fluorochrome is not visible to the human eye and therefore cost-prohibitive with imaging systems costing around $100,000 [26]. Furthermore, 5-ALA showed modest specificity due to the decreasing fluorescence in the infiltrative margin. This shortcoming might be resolved by combining 5-ALA with fluorescein. This combination better illuminates the tumour by appearing orange-to-red in tumour tissue and green in normal tissue [80]. A combination of fluorescein and 5-ALA with iMRI showed a significant increase in the extent of resection as compared to iMRI alone, with up to 100% gross total resection in the contrast-enhancing tumours. However, the incidence of postoperative neurological deficits was not lower [81]. the applicability of iMRI is further limited due to high costs and increased operative times. Besides, 5-ALA is often used in combination with iUS as an inexpensive technique that is widely available and allows better delineation of the tumour. In turn, iUS can be integrated into neuronavigation to compensate for brain-shift. Ultimately, the used combinations must be tailored to the individual patient. For surgery of tumours involving eloquent areas the determination of tumour margins is not enough and precise brain mapping is essential to allow a safe resection. Currently, neuronavigation, combined with the preoperative functional MRI, is often considered a gold standard since it allows the surgeon to plan and visualize the operative path to the target and the brain functional localization [82]. However, functional MRI does not distinguish structures essential for functional preservation. Instead, Intraoperative neuromonitoring might allow localisation of these eloquent areas and might reduce post operative dysfunction, significantly improving the quality of live. Examples of currently used intraoperative neuromonitoring techniques are somatosensory evoked potential (SSEP), direct electrical stimulation (DES), motor evoked potential (MEP), electromyography (EMG), and electrocorticography (ECoG) [83].

**Table 4:** Strengths and limitations of the different modalities

Technique	Sensitivity (%)	Specificity (%)	Label-free	Label administration time (min)	Detection time (min)	Additional cost	Usability	Applicable for multiple pathologies
5-ALA	89.0	84.6	No	120–240	0	Low	High	Yes
Fluorescein	84.8	86.9	No	0.12–0.23	0	yes	–	No
Indocyanine green	95.4	55.2	No	900	0	High	High	No
Neuronavigation	57.8	57.4	Yes	–	1–2	Low	High	Yes
iMRI	72.1	75.4	Yes	–	15–60	High	Low	No
iUS	53.7	87.2	Yes	–	1–2	Low	High	Yes
DESI	86.0	93.2	Yes	–	3	High	Low	Yes
MALDI	–	–	Yes	–	> 30	High	Low	Yes
REIMS	96.4	100	Yes	–	0.01–0.04	High	High	Yes
Raman spectroscopy	93.8	96.5	Yes	–	0–0.03	Low	Low	No
Hyperspectral imaging	61.4	91.8	Yes	–	1	Low	Low	No

Sensitivity and specificity results represent the weighted mean % (taking into account the number of patients participating in the study) as calculated over the investigated studies. Additional costs refer to the necessity of high instrumental or maintenance costs and for usability, the level of expertise was evaluated

*5-ALA* 5-aminolevulinic acid, *iMRI* intraoperative magnetic resonance imaging, *iUS* intraoperative ultrasound, *DESI-MS* desorption electrospray ionisation mass spectrometry, *MALDI* matrix assisted laser desorption ionization, *REIMS* rapid evaporative ionisation mass spectrometry

The creation of Raman spectroscopy and hyperspectral imaging enabled for label free-imaging of unprocessed tissue. This allowed detection of infiltrative cells at the margin and quantification of the PpIX fluorescence at cell level [24]. However, none of these techniques offer the molecular precision to delineate the infiltrative margin and the extent of resection. Mass spectrometry-based techniques, such as DESI-MSI, MALDI-MSI and REIMS, have a rather high instrumental purchase cost, but detect the complete mass range of biomolecules, including biomarkers such as proteins and lipids. the ambient ionization methods, DESI-MSI and REIMS, allow real-time feedback, without the need of sample preparation. They provide a more sensitive platform to characterize the tumour and its margin by identifying changes in the molecular profile of the investigated tissue. Furthermore, a multimodal mass spectrometry approach should be evaluated, using MSI to visualise and localize, combined with REIMS to identify key compounds specific to gliomas. Thanks to the specific research capabilities of these techniques, tumour tissue can directly be identified without the use of labelling compounds or tracers. Moreover, detailed spatial information can be obtained. The visualization of interesting compounds will allow to obtain insight into heterogenous tumours, even up until the cellular level. Thanks to the combination of visualization (MALDI) and a very fast chemical profile (REIMS), a reliable tissue recognition system can be built and used during glioma surgery. Ultimately, in combination with the standard image guided techniques, these mass spectrometry-based diagnostics might not only serve to guide tumour resection, but might also expand our understanding of brain tumours.

## Discussion

The ultimate goal of brain tumour surgery is to maximize the extent of brain tumour resection, while preserving neurological functions and guaranteeing patient safety. Over the years, multiple techniques have been developed and evaluated for the detection and identification of tumour tissue. All showed different levels of accuracy, sensitivity and specificity for the different types and grades of brain tumours. Therefore, it is important to remember that resection in eloquent brain areas cannot be guided by images of fluorescence alone. Surgery of brain tumours should always be guided by the competence of the surgeon to associate the surgical anatomy with auxiliary techniques. This review describes the diagnostic accuracy of conventional and experimental techniques for glioma surgery. Special attention was paid on the invasive margin of these infiltrative tumours and the intra-operative, real-time differentiation between tumour and normal tissue.

We can conclude that all investigated methods had methodological and practical limitations, that guided researchers towards the combined use of intraoperative imaging techniques. This often showed an improved outcome in terms of extent of tumour resection and progression free survival of the patients. As more technologies are incorporated into the operating room, neurosurgeons are able to select the most appropriate technique based on the tumour characteristics and their expertise with the different technologies. Here, we are confident that with the necessary large randomized-controlled clinical trials and technological advancements intraoperative tumour detection will become an essential part in the surgical resection of brain tumours.

## Data Availability

Not applicable. All data used in this review was referred to in the Reference section.
